# Probability of ventricular fibrillation: allometric model based on the ST deviation

**DOI:** 10.1186/1475-925X-10-2

**Published:** 2011-01-13

**Authors:** Maria P Bonomini, Pedro D Arini, Max E Valentinuzzi

**Affiliations:** 1 Instituto de Ingeniería Biomédica (IIBM), Facultad de Ingeniería (FI), Universidad de Buenos Aires (UBA), Buenos Aires, Argentina; 2 Instituto Argentino de Matemática (IAM) "Alberto Calderón", Consejo Nacional de Investigaciones Científicas y Técnicas (CONICET), Buenos Aires, Argentina

## Abstract

**Background:**

Allometry, in general biology, measures the relative growth of a part in relation to the whole living organism. Using reported clinical data, we apply this concept for evaluating the probability of ventricular fibrillation based on the electrocardiographic ST-segment deviation values.

**Methods:**

Data collected by previous reports were used to fit an allometric model in order to estimate ventricular fibrillation probability. Patients presenting either with death, myocardial infarction or unstable angina were included to calculate such probability as, *VF*_*p *_*= δ + β (ST)*, for three different ST deviations. The coefficients *δ *and *β *were obtained as the best fit to the clinical data extended over observational periods of 1, 6, 12 and 48 months from occurrence of the first reported chest pain accompanied by ST deviation.

**Results:**

By application of the above equation in log-log representation, the fitting procedure produced the following overall coefficients: Average *β *= 0.46, with a maximum = 0.62 and a minimum = 0.42; Average *δ *= 1.28, with a maximum = 1.79 and a minimum = 0.92. For a 2 mm ST-deviation, the full range of predicted ventricular fibrillation probability extended from about 13% at 1 month up to 86% at 4 years after the original cardiac event.

**Conclusions:**

These results, at least preliminarily, appear acceptable and still call for full clinical test. The model seems promising, especially if other parameters were taken into account, such as blood cardiac enzyme concentrations, ischemic or infarcted epicardial areas or ejection fraction. It is concluded, considering these results and a few references found in the literature, that the allometric model shows good predictive practical value to aid medical decisions.

## Background

Ventricular fibrillation can be viewed as a probabilistic event that appears biased under certain pathophysiological and daily life situations. Physicians, in their practice, try to predict as close as possible how high such probability is. Since most of cardiac deaths are due to ventricular fibrillation, it can be said that such death, in the end, would be an event that counts as a ventricular fibrillation. Valentinuzzi, in 2010, has reviewed at large such arrhythmia and its countermeasures [1]. Empirical tests, as possible quantitative criteria to screen out patients of high risk (that is, searching for a better answer to the question *shall we confine the patient to the coronary unit*?) have been attempted with moderate success, but always the degree of uncertainty is rather large. In such endeavor, we might try an appealing and old universal scaling, the allometric law, although in principle apparently not related to the cardiac risk overall concept, it might find a place in it and at least deserves to be reminded bringing about first a nice and well carried out paper by Noujaim *et al*, in 2004 [2]. In it, it is recalled that from mouse to whale the electrocardiographic PR interval increases 10^1 ^times whereas body mass (*B*_*M*_) augments 10^6^. This is the first use we found of the allometric law in cardiology encouraging us to proceed further up.

Scaling of many biological processes can be described by the allometric equation, *Y *= *a*(*B*_*M*_)^*b*^, where *Y *is the biological process and *a *and *b *are scaling constants. In general, the weights of most individual organs scale as a constant fraction of body mass (i.e., the body mass exponent, *b *equals 1.0). Biological rates (heart rate, respiratory rate) scale as *b *close to 0.25. Finally, volume rates (the product of volume and rate) such as cardiac output, ventilation and oxygen uptake vary as *b *around 0.75. These emergent patterns provide insights into body-size dependent 'principles of design' that seem to dictate several blueprint aspects and function across species among all mammals [3].

Noujaim *et al *[2] assumed that the heart behaves as a set of "fractal-like" networks tending to minimize propagation time across the conducting system while ensuring a hemodynamically optimal atrioventricular activation sequence. With the potential relationship given above and, subsequently, based on previously published values of PR interval, heart rate, and body masses of 541 mammals, they reported as best fit the equation *PR *= 53(*B*_*M*_)^0.24^.

Inspired in the latter report, the following question seems pertinent: Would a relationship similar to the allometric equation be conceivable, say, between the probability of cardiac risk (or cardiac event or episode, all equivalent terms) and heart weight, or perhaps other parameter somehow related to the latter, as for example, the number of cardiac diseased fibers or the ST shift seen in the ECG? The objective of this communication tries to find an answer to such question. The mentioned ECG deviation appears as a good candidate because well-known is the fact that the larger the ST step, in either direction (upward or downward), the larger the compromised myocardial mass. In some cases, such change includes inverted T-wave, which tends to complicate the wave-pattern. Many reports confirm this concept, such as Klootwijk, in 1998 [4], Kléber, in 2000 [5], or Balian *et al*, in 2006 [6], among others, where often the ST shift is defined as a change of ST amplitude in one or more leads of at least ± 100 μV from the baseline ST level, developing within a 10 minute period and persisting for at least 1 minute. However, differences among authors regarding these criteria are frequent.

## Methods

### Theoretical background

Allometry, in general biology, measures the relative growth of a part in relation to the whole living organism. The term was first used by Snell, in 1891 [7], to express the mass of a mammal's brain as a function of the body mass. The growth velocity of a component *y *is related to the growth velocity of another component (or the whole organism) *x *in a constant way. This was clearly described by von Bertalanffy in 1957 [8]. Thus, the relative rate of change of a given event *y *is proportional to the relative rate of change of body mass or body weight *x*, i.e.,

(1) $$\frac{dy/dt}{y}=B\frac{dx/dt}{x}$$

After integration and some easy algebraic manipulation, equation (1) becomes

(2) lny=lnA+Blnx

or

(3) $$y=Ax^{B}$$

Originally, *y *was the weight of an organ (heart, stomach, other) and *x *was body weight or mass. The parameters *A *and *B *require numerical estimation by an appropriate procedure usually using empirical information. By the same token, let us say that the probability of fibrillation *P*_*F *_(and we use *P*_*F *_because, as stated above, most of the cardiac episodes end up in ventricular fibrillation) follows a relationship with the number of ventricular diseased fibers (*N*_*DF*_) formally equal to (2), i.e.,

(4) $$P_{F}=\alpha {\left(N_{DF}\right)}^{\beta }$$

Hence, *y *in equation (3) is replaced by *P*_*F *_in (4), and *N*_*DF *_in the latter takes the place of *x *in the former. After all, the number of diseased cardiac fibers (ischemic or infarcted or both) are part of the cardiac mass. Besides, since the electrocardiographic ST-segment deviation (Δ_*ST*_) is a traditional estimator of cardiac injury, it sounds sensible to state that,

(5) $$N_{DF}=\gamma \Delta_{ST}$$

or in words, the number of diseased ventricular fibers is proportional to the ST-deviation (Δ indicating precisely "deviation"). Hence,

(6) $$P_{F}=\alpha {\left(\gamma \Delta_{ST}\right)}^{\beta }$$

After taking logarithms of both sides, the latter equation becomes,

(7) $$\ln P_{F}=(\ln\alpha +\beta \ln\gamma )+\beta (\ln\Delta_{ST})$$

which can be reduced to,

(8) $$VF_{P}=\delta +\beta (ST)$$

We define *VF*_*P *_as ventricular fibrillation probability, where

(9) δ=lnα+βlnγ

(10) $$ST=\ln\Delta_{ST}$$

and

(11) $$VF_{P}=\ln P_{F}$$

Hence, equation (8) in log-log plot would represent the probability of fibrillation as function of the ECG ST-depression or elevation.

### Numerical procedure

To calculate out the two constants *δ *and *β *of equation (8) and later on apply the mathematical expression for predictive purposes, the probability of the data having occurred can be estimated by, (a) simply assuming an arbitrary and theoretical set of coupled pairs of numbers, as for example, a quadratic law of the type w = K z^2^, that is, the *VF*_*P *_would be accepted as being proportional to the square of the ST, or (b) using a particular hypothesis, say, based on clinical data.

Medical experience is obviously the best and most reliable source of information where from an idea of the probability of fibrillation based on ECG evidence can supply an excellent lead. For that matter, three sets (i, ii and iii) were used to fit the allometric equation, two from Hyde *et al *[9] and another from Kaul *et al *[10], as follows:

(i) In the first one, 642 patients had been admitted to coronary care unit with prolonged chest pain. Due to the exclusion criteria applied by these authors, 469 were removed leaving a net number of 173 for their study. Besides, they reported survival rates at 1 and 4 years after the first admission.

(ii) In the second paper (PARAGON-A trial), out of 2,282 patients with chest discomfort within the previous 12 hours, there was a screen out of 694 due to either missing or not clear enough records leaving a net of 1,588 cases. They were evaluated at 1 month and 6 months.

(iii) Besides, the latter authors had 8,001 patients (GUSTO-IIb trial) comparing hirudin and heparin therapy when unstable angina or acute myocardial infarction was present without ST-segment. Out of this total, only 6,301 were evaluated at 1 month and 6 months.

In Hyde *et al *[9], patients with ≥ 0.5 mm ST-segment depression were classified as "true depression". This deviation was subclassified as 0.5 mm, 1 mm or ≥ 2 mm. In their own words, "ST segment depression was measured using calipers 80 ms after the J point in intervals of 0.5 mm. ECGs were analyzed blinded to the clinical outcome".

The ST-segment criteria in Kaul *et al *[10], instead, rounded out the depression of 0.5 mm to 1 mm, of 1.5 mm to 2 mm, including in the latter larger deviations, distinguishing three groups: No ST-segment depression, 1 mm ST-segment depression in two contiguous leads, and ST-segment depression of 2 mm in two contiguous leads. The 12-lead ECGs were recorded at a paper speed of 25 mm/s. ST segment depression was judged to be present if the J point was depressed by ≥1 mm and was followed by a horizontal or downsloping ST segment for at least 0.08s in one or more of the 12 leads, except for the aVR lead.

Curves presented herein were constructed after the numerical values given in [9,10]. All were resampled with a quadratic interpolation function in steps of 0.025 mm to improve the resolution. Thereafter, a log-log algorithm was applied to the ventricular fibrillation probability versus the ST-segment deviation (see equation 6). The parameters *β *and *δ *and goodness of fit *r*^*2 *^were computed by linear regression and all quadratic fits used values within the 0.5-2 mm range (see Table 1). It should be recalled that the standard ECG calibration of 10 mm = 1 mV is used in all the paper.

**Table 1:** Coefficients *β *and *δ*, in equation (6), and adjusted r-square for all 9 curves.

	Months	*β*	*δ*	Adjusted *r*-square
**PARAGON-A**	1	0.42	1.09	0.9884
**Kaul *et al***. [2] **(corresponding to Figure 1)**	6	0.45	1.23	0.9917

**GUSTO-IIB**	1	0.62	0.92	0.9245
**Kaul *et al***. [2] **(corresponding to Figure 1)**	6	0.54	1.09	0.9427

**Mean PARAGON-A & GUSTO- IIB**	1	0.50	1.01	0.9615
**Kaul *et al***. [2] **(corresponding to Figure 2)**	6	0.49	1.16	0.9734

	12	0.54	1.56	0.9844
**Hyde *at al***. [1] **(corresponding to Figure 1)**	48	0.48	1.79	0.9477

**Average curve (corresponding to Figure 3)**	1 to 48	0.46	1.28	0.9959

## Results

**Figure 1:**
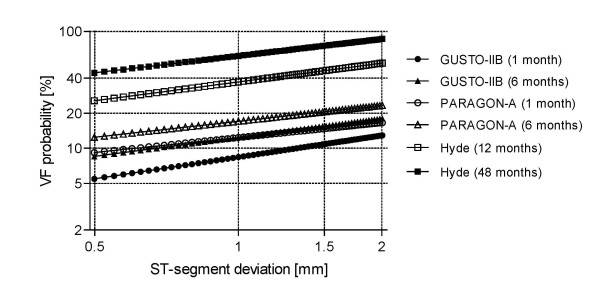
**Estimated *VF*_*p *_as function of ST shift at different observational windows**. All 6 curves drawn after data from references Hyde *et al *and Kaul *et al *[1,2], at 1, 6, 12 and 48 months (see text for details). For a 2 mm ST-deviation, the full range of predicted risk probability extends from about 12.8% at 1 month up to 83.5% at 48 months after the original cardiac event.

Figure 1 displays all 6 curves, where there are 2 clearly distinguishable groups: The lower one corresponds to 1 and 6 months after confinement, as reported by the GUSTO-IIb data (dark markers) and by the PARAGON-A study (open markers), both in the same paper of Kaul *et al *[10]. As example, for 1.5 mm shift, the predicted probability at one month ranges from slightly below 11% (GUSTO-IIb) to 15% (PARAGON-A). For the same ST shift selected above, the probability values at 6 months span from 15% (GUSTO-IIb) to a 20% (PARAGON-A). Finally, the upper two curves describe the behavior at 1 (open squares) and 4 years (dark squares) after the event, according to Hyde *et al *[9]. For the same previous ST deviation, the foreseen probability range goes from 45% to about 75%. The fitted adjustments pass essentially through the depicted points.

**Figure 2:**
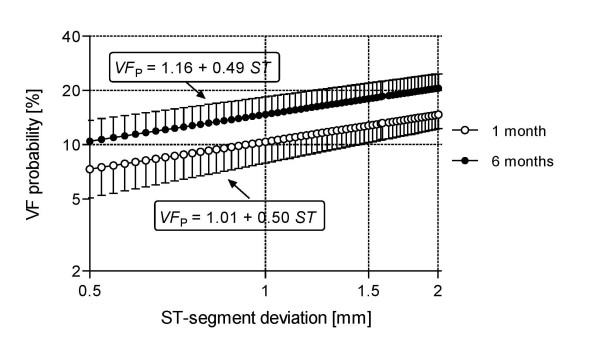
**Average *VF*_*p *_from two studies**. Mean ± SD results, obtained from mean PARAGON-A and GUSTO-IIB.

In Figure 2, instead, we collect the results after averaging out PARAGON's and GUSTO's data, as reported by Kaul *et al *[10], respectively, at 1 and 6 months, from bottom to top, showing also one half of the standard deviation for each data point. Notice the spread decrease comparing the bottom with the upper curve at any ST value. Besides, an inverse relationship between ST deviation and standard deviation is manifest, which speaks of the gradual nature of ST changes and, therefore, points out to the importance of such amplitude.

**Figure 3:**
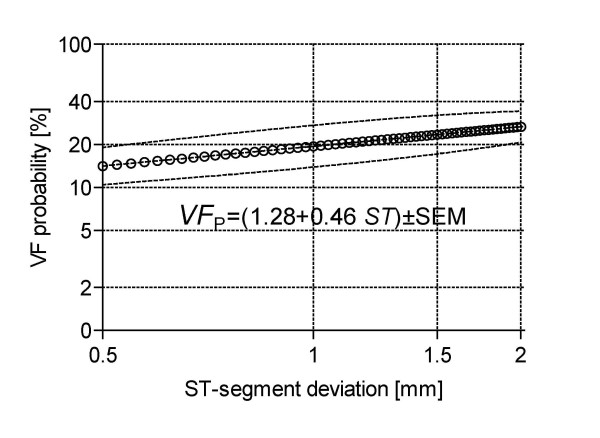
**Overall average curve covering all the reported data**. The upper and lower dashed lines bound the SEM so giving an idea of the possible error in the prediction. Say, for 1.5 mm ST-segment shift, the VF probability would go from 17% to 32%.

Figure 3 is an attempt to reach a single equation for all the data presented in Figure 1. For that matter, an average value curve is depicted along with its Standard Error of the Mean (SEM). Dispersion here covers the full time range, i.e., from 1 month to 4 years. Table 1 summarizes the numerical values for the two parameters characterizing equation (8).

## Discussion

This report has developed an allometric equation simply based on the electrocardiographic ST-segment deviation. The calculated coefficients permit predictions at different times after the first cardiac episode or, with a much wider spread, as an overall quantitative evaluation applying the relationship given in Figure 3. Obviously, the model must be tested in the clinical environment to better assess its accuracy and predictive power. In Figure 2, dispersion increases at 1 month after the cardiac episode, which might be interpreted as the patient still traversing a period of dangerous instability. Conversely, the upper curve, after a longer time, shows a marked spread decrease. We read this fact as a stable condition because of compensation.

With the aging process, along perhaps with an increase in the ischemic areas or deterioration of the myocardial scar tissue, it seems quite acceptable a consequent increase, too, in the probability of an arrhythmic event, as well depicted in the three figures, throughout longer observational periods.

It is convenient to underline that the best fit quadratic equation (see Methods) supplies the numerical information needed to estimate the *β *and *δ *constants of the allometric law.

This model uses only the ST-segment as criterion, which obviously leaves out other possible parameters, such as myocardial enzymes (CPK, for example), quantitatively obtainable by blood sample analysis, or ischemic or infarcted epicardial surface, from appropriate imaging procedures, or ejection fraction as evaluated by echocardiography. Other anthropological data, such as patient's sex and age, could also be included. Any of these criteria would lead to allometric equations as the one herein reported. One tempting and difficult approach would try to combine all the mentioned parameters in a single mathematical model.

The results herein presented foresee a direct application in the clinical environment to better predict the evaluation of a cardiac patient. However, this kind of validation remains to be carried out.

## Conclusions

The allometric statement seems to maintain interest, especially in general mammalian biology [11,12] and the results reported here would indicate an attractive line of research with their consequent clinical tests. Once more, it should be underlined the proportion basis of the allometric statement, since it numerically links here a specific number of compromised fibers (ischemic or even dead) with the concept of cardiac risk.

## Competing interests

The authors declare that they have no competing interests.

## Authors' contributions

All three authors have approximately contributed in equal proportions having exchanged ideas and opinions in several working sessions. Calculations and graphs were mainly made by PDA and MPB while the text draft was mostly in the hands of MEV. All authors read and approved the final manuscript.
